# Importance Of Thorough Physical Examination: A Lost Art

**DOI:** 10.7759/cureus.1212

**Published:** 2017-05-02

**Authors:** Talal Asif, Amena Mohiuddin, Badar Hasan, Rebecca R Pauly

**Affiliations:** 1 Department of Internal Medicine, University of Missouri Kansas City (UMKC)

**Keywords:** physical examination skills, infective endocarditis, central facial nerve palsy, embolic stroke

## Abstract

Several recent studies have described a deterioration in physical examination skills among modern physicians. Reasons hypothesized for this change are improvements in technology and time constraints. Poor physical exam skills are a noteworthy threat to patient safety as they can lead to incorrect as well as missed diagnoses, causing delays in timely implementation of life-saving treatments. Here, we present a case of extensive acute embolic strokes secondary to infective endocarditis. The patient was initially misdiagnosed as having Bell’s palsy due to incorrect physical examination. Through this case, we highlight the importance of management guided by a thorough history and physical examination to minimize diagnostic errors.

## Introduction

Alarmingly, several recent studies document the decline in physical examination skills among physicians [1]. Reasons postulated for this shift in approach are improvements in technology, time constraints, and uncertainty that stems from a lack of confidence in physical exam skills [1]. Increased reliance on laboratory investigations and imaging has generated several problems that are a significant threat to patient safety. These include incorrect as well as delayed diagnosis, leading to gaps in the implementation of life-saving treatments. Here, we present a case of extensive acute embolic strokes secondary to infective endocarditis that was initially misdiagnosed as Bell’s palsy due to sparse and incorrect physical examination. Through this case, we aim to advocate for the use of thorough and differential diagnosis directed history and physical examination to guide appropriate workup.

## Case presentation

**Figure 1:**
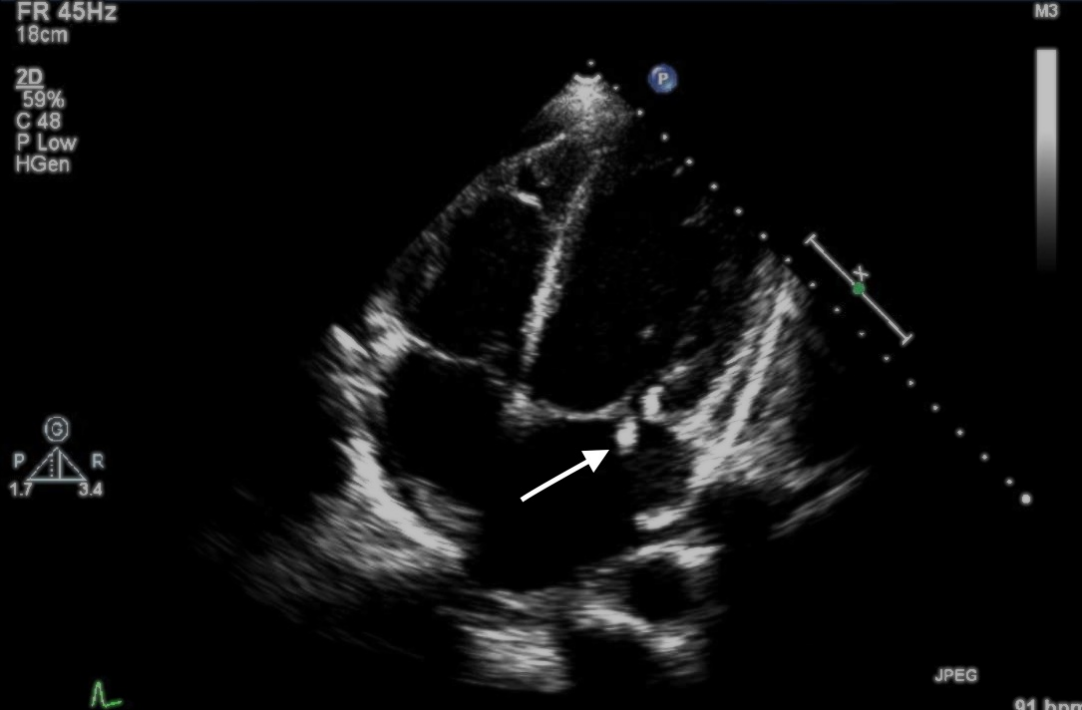
Transthoracic echocardiogram (TTE) showing mobile echodensity (arrow) on the mitral valve leaflet measuring 1.5 x 1.3 cm

A 28-year-old male patient with a history of substance abuse presented to the emergency department with a one-day history of a sudden onset left facial paralysis. Computed tomographic (CT) scan of the head showed no acute intracranial process. The patient was diagnosed with Bell’s palsy, started on oral steroids, and discharged. The next day, he returned with left arm weakness. The admitting team was asked to evaluate the patient; physical examination revealed sparing of the forehead muscles on the affected side, which sparked our suspicion that the original diagnosis of Bell’s palsy may have been in error. Power in the left upper extremities was 3/5 as compared to 5/5 on the right. The patient had a pansystolic murmur over the mitral area with splinter hemorrhages and Janeway lesions in lateral toes. On meticulous history, the patient admitted to daily intravenous heroin injections, having strongly denied this at first. Neurology and cardiology teams were asked to evaluate the patient emergently. A transthoracic echocardiogram (TTE) revealed a 1.5 x 1.3 cm vegetation on his mitral valve (Figure 1).

**Figure 2:**
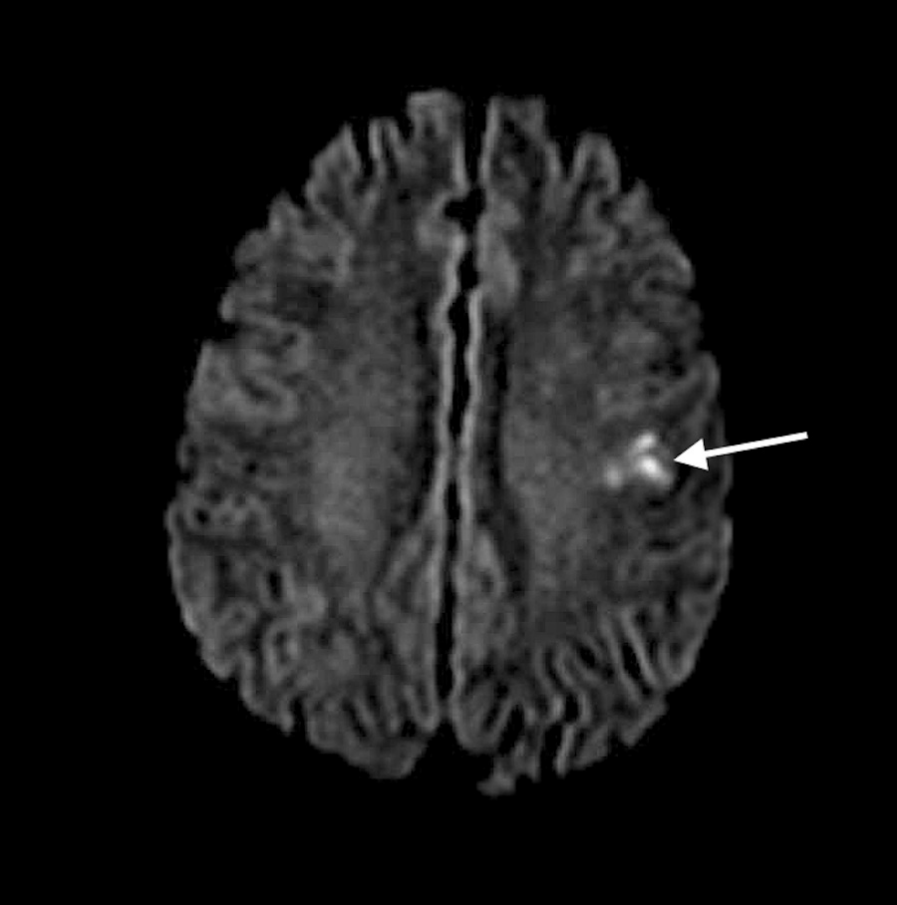
Magnetic resonance imaging (MRI) of the brain showing area of diffusion restriction (arrow) in the left parietal region

Magnetic resonance imaging of the brain showed multifocal areas of diffusion restriction throughout both cerebral hemispheres consistent with acute infarcts (Figure 2).

**Figure 3:**
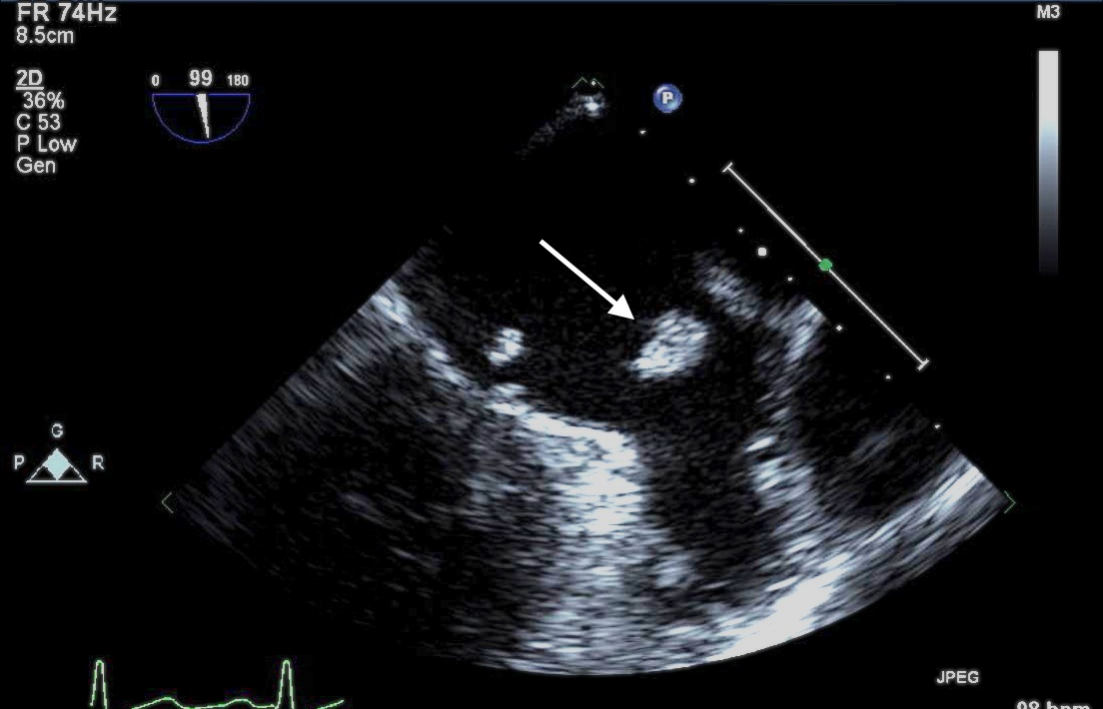
Transesophageal echocardiogram (TEE) showing the mitral valve vegetation (arrow)

Blood cultures were obtained, and the patient was started on broad-spectrum intravenous antibiotics and transferred to the cardiac intensive care unit for further management. Blood cultures eventually returned positive for viridans streptococci, and he was treated with six weeks of intravenous ceftriaxone. Transesophageal echocardiogram (TEE) was also obtained that verified the findings seen on TTE (Figure 3).

He underwent emergent mitral valve replacement surgery to prevent recurrent embolic strokes.

## Discussion

Shortcomings in physical examination skills among residents and faculty have been documented in numerous studies due to the decline in use and an increased dependence on technology [2]. It is estimated that hospitalists spend less than 18% and internal medicine interns less than 12% of their time in direct patient care [3-4]. Poor physical examination skills are a threat to patient safety as the probability of diagnostic errors and oversights is increased [5]. Moreover, unnecessary investigations themselves are potentially harmful [2]. In an era where there is growing concern of over-utilization of health care resources and expense, poor physical examination skills lead to more injudicious referrals and patient mismanagement, leading to added costs. The unnecessary reliance on investigations has made it harder for modern-day physicians to meet the day-to-day needs of patients seeking medical care, especially in resource-limited settings [6].

To make matters worse, this issue has received insufficient attention. Promotion of modern diagnostic technology, a lack of bedside teaching, and decreased interest in physical examination owing to time limitations has led to the further neglect of physical examination competence [7]. Our case aims to raise awareness of this decay in clinical skills and its probable negative impact on patient outcomes. For instance, our patient was originally diagnosed with Bell’s palsy. Classically, Bell’s palsy causes weakness of the entire unilateral face, including the failure of forehead muscle wrinkling on the affected side. However, in actuality, there was sparing of the forehead muscles on the affected side in our patient, owing to the bilateral innervation of these muscles. This generally should have prompted suspicion of a central nervous system lesion, such as stroke. The resulting delay in diagnosis contributed to patient morbidity, an unfortunate consequence that could have been avoided with a good physical exam. A thorough physical examination itself is certainly not a substitute for the use of technology. However, we encourage the appropriate application of a thorough history and physical examination to guide the prudent use of technology. Potential advantages of this approach include enhanced physician-patient relationship, improved patient safety, fewer diagnostic errors, and lower financial costs [2].

The perfection of physical examination skill requires continued efforts and practice to increase its diagnostic yield [7-8]. We predict that the greatest benefit will be obtained through interventions targeted at medical students and residents as physicians deliver health care the way they have been taught [9]. Through this case, we also aim to create mindfulness among young physicians that focus on clinical skill needs to begin during the learning phase of their careers.

## Conclusions

This case demonstrates that indiscriminate use of technology does not translate into comprehensive high quality or safe patient care. A proper choice of investigations guided by logical clinical decision-making after integrating the clinical history and physical exam in differential diagnosis is imperative for timely diagnosis to enhance patient safety.
